# No evidence of a role of the β4 subunit of the nicotinic acetylcholine receptor in alcohol-related behaviors

**DOI:** 10.1186/s13104-017-2470-7

**Published:** 2017-04-05

**Authors:** Helen M. Kamens, Constanza Silva, Riley McCarthy, Ryan J. Cox, Marissa A. Ehringer

**Affiliations:** 1 Department of Biobehavioral Health, Penn State University, University Park, PA USA; 2 Center for Brain, Behavior, and Cognition, Penn State University, University Park, PA USA; 3 Department of Integrative Physiology, University of Colorado, Boulder, CO USA; 4 Institute for Behavioral Genetics, University of Colorado, Boulder, CO USA

**Keywords:** Ethanol, Nicotinic acetylcholine receptors, Ataxia, Sedation, Consumption, *Chrnb4*

## Abstract

**Background:**

Nicotinic acetylcholine receptors have gained attention in the last several years as mediators of alcohol-related behaviors. The genes that code for the α5, α3, and β4 subunits (*Chrna5*, *Chrna3*, and *Chrnb4*, respectively) map adjacent to each other on human chromosome 15/mouse chromosome 9. Genetic variants in this region have been associated with alcohol phenotypes and mice that overexpress these three subunits have reduced ethanol intake. In the present experiments, we examined the role of the *Chrnb4* gene in three ethanol behaviors: consumption, ataxia, and sedation. Wildtype, heterozygous, and knockout mice were tested for ethanol consumption with a 2-bottle choice procedure and the drinking-in-the-dark paradigm. Ethanol-induced ataxia was measured with the balance beam and dowel test. Finally, the sedative effects of ethanol were measured with the loss of righting reflex paradigm.

**Results:**

We observed no significant genotypic effects on any of the ethanol behaviors examined, suggesting that the β4 subunit is not involved in mediating these responses.

**Conclusions:**

While we found no evidence for the involvement of the β4 subunit in ethanol responses, it is possible that this subunit modulates other behaviors not tested and further work should address this before completely ruling out its involvement.

**Electronic supplementary material:**

The online version of this article (doi:10.1186/s13104-017-2470-7) contains supplementary material, which is available to authorized users.

## Background

In the United States, the lifetime prevalence of having an alcohol use disorder is approximately 29%, and any given year about 14% of the population meets DSM-5 criteria [1]. Use of alcohol leads to high morbidity and mortality [2]. Unfortunately, current medications available to treat alcoholism fall short of helping those suffering from alcohol use disorders. These data provide a strong rationale for the need to identify new targets for possible pharmacotherapies for alcoholism. Neuronal nicotinic acetylcholine receptors have been identified as possible targets, since drugs that act at these receptors show potential as a therapeutic option for alcoholism [3, 4].

Nicotinic acetylcholine receptors are ligand gated ion channels. These receptors are comprised of five subunits around a central pore and allow the flux of cations across the membrane when stimulated [5, 6]. Two varieties of nicotinic receptors are found in mammalian cells: homomeric receptors composed of five of the same subunit (e.g. the α7 receptor) and heteromeric receptors comprised of α and β subunits (e.g. the α4β2 receptor). Nicotinic receptors are distributed widely throughout the brain including in regions known to influence drug behaviors [7].

Human genetic studies have implicated nicotinic acetylcholine receptors in a number of alcohol-related behaviors. The genes encoding the α5, α3, and β4 subunits (*CHRNA5*, *CHRNA3*, and *CHRNB4*, respectively) are located adjacent to each other on human chromosome 15/mouse chromosome 9. Variants in this gene cluster have been associated with a number of alcohol-related behaviors including: the initiation of alcohol consumption [8], level of response to alcohol [9, 10], alcohol use [11], and dependence [12]. When this gene cluster was overexpressed in a transgenic mouse model, reduced ethanol consumption was observed [13]. Unfortunately, all three subunits were overexpressed, thus the specific subunit(s) contributing to this effect is unknown. Pharmacological studies provide support for the role of α3β4 receptors in ethanol self-administration wherein rats treated with a partial agonist of this receptor displayed reduced ethanol self-administration [14]. However, to date the specific involvement of the β4 receptor subunit in alcohol-related behaviors has not been studied.

To examine the role of *Chrnb4* in ethanol responses we tested mice lacking this subunit. Mice were tested in five behavioral assays. Voluntary ethanol consumption using a standard two-bottle choice paradigm, binge-like ethanol intake with the drinking-in-the-dark (DID) protocol, ethanol-induced ataxia using the balance beam and dowel test, and ethanol-induced sedation using the loss of righting reflex (LORR) paradigm. We hypothesized that mice lacking the *Chrnb4* gene would display altered responses to ethanol.

## Methods

### Animals

Male and female wild-type (WT), heterozygous (HET), and knockout (KO) animals used in these experiments were bred at the Institute for Behavioral Genetics animal facility. Mice entered into testing between 2 and 4 months of age (ages and weights of experimental mice are presented in Additional file 1). Mice deficient in *Chrnb4* were previously generated using homologous recombination technology by introducing a 4.1 kb deletion into the gene that included a disruption of exon 5 [15]. This mutation has been backcrossed to C57BL/6 mice for at least 10 generations and resulting animals have been maintained at the University of Colorado through HET breeder pairs. This breeding scheme allowed for littermates to be tested in this study. Genotypes were determined from DNA extract from tail samples based on published parameters [16]. Briefly, genotypes were determined by a touch down PCR reaction with the following primers: forward, 5′-TGTAGAGCGAGCATCCGAACA-3; β4 wild-type reverse, 5′-TCTCTACTTAGGCTGCCTGTCT-3′; and β4 mutant reverse, 5′-AGTACCTTCTGAGGCGGAAAGA-3′. Animals were housed 1–5 per cage in standard mouse caging (29.2 × 19.1 × 12.7 cm) lined with Bed-o’Cobs. Mice had ad libitum water and rodent chow (5001 Lab Diet^©^) unless noted below. The animal colony was maintained on a 12-h light/dark cycle (lights on at 0700 h). Behavioral experiments were performed between 0900 and 1700 h. The University of Colorado’s Institutional Animal Care and Use Committee approved all testing. The University of Colorado complies with the Animal Welfare Act and Federal regulations on the use of animals in research.

### Drugs

Ethyl alcohol (200 proof; Pharmco, Brookfield, CT, USA) was used for all experiments. The ethanol was diluted in physiological saline (0.9% NaCl) for i.p. injections (20% v/v) or tap water for drinking solutions. Saccharin sodium salt, quinine hemisulfate salt, and sucrose were obtained from Sigma-Aldrich (St. Louis, MO, USA) and diluted in tap water to appropriate concentrations.

### Two-bottle choice ethanol consumption

Choice ethanol consumption was measured in 25 female *Chrnb4* mice using the 2-bottle free choice paradigm [17–19]. Mice were singly housed and presented with two glass bottles fitted with drinking spouts. Mice were allowed to habituate to this test environment for 2 days, during this time both bottles contained water. On the first day of the experiment (Day 1), one of the water tubes was replaced with a tube containing 3% ethanol. The side of the cage that the ethanol bottle was presented was switched every 2 days. Every 4 days the concentration of ethanol increased (3, 7, 10, and 20%). Two control cages (no animal) were handled similar to the experimental cages. Measurements from tubes on these control cages were used to quantify evaporation/leakage, and individual drinking values were corrected with these data. Stable consumption for each ethanol concentration was measured by the average of days 2 and 4 and used for the analysis [20]. Three dependent variables were obtained: average 24 h ethanol consumption (g/kg), ethanol preference (ml of ethanol/total ml fluid), and total volume consumed (ml).

### Two-bottle choice tastant consumption

One week following the completion of the 2-bottle choice ethanol consumption study, the same mice were tested for the consumption of sweet (saccharin) and bitter (quinine) tastants [17, 21]. Procedures were identical to that for choice ethanol consumption except that mice were offered the choice of water and a tastant solution. Both saccharin (0.033 and 0.066%) and quinine (0.015 and 0.03 mM) were tested in all animals. Each tastant was presented for 4 days starting with the lower concentration. Fluid levels were read each day and bottles switched sides every 2 days. Dependent variables included: average 24 h tastant consumption (mg/kg), tastant preference (ml of tastant/total ml fluid consumed), and total fluid consumption (ml).

### Drinking-in-the-dark (DID)

To determine the effect of *Chrnb4* on binge-like ethanol consumption the DID paradigm was used [22, 23]. Briefly, 34 naïve male and female mice were acclimated to a reverse light/dark cycle (lights on at 2200) for at least 2 weeks prior to testing. Mice were singly housed during the final 5 days of acclimation prior to the 4 day DID procedure. On days 1–3, 3 h after lights off, the water bottle was removed and replaced with a single bottle containing 20% ethanol. Animals had access to ethanol for 2 h before the tube was removed and replaced with water. On day 4 the ethanol tube was available for 4 h. At the end of the 4 h drinking session, a 10 µl blood sample was taken from the tail vein for analysis of blood ethanol concentrations (BEC) based on previously published methods [24]. Following the completion of the ethanol DID procedure, the mice were left undisturbed for 1 week before they underwent the same procedure, but 10% sucrose was available during the testing [25].

### Balance beam

The balance beam test was used to assess ethanol-induced ataxia [26, 27]. Using published procedures, 50 naïve male and female *Chrnb4* mice were tested on a 104.1 cm long by 1.9 cm wide, PVC beam. The balance beam was elevated 54.6 cm from the floor. Mice underwent a training session during which they had to cross the beam two times. To measure baseline ataxia, the number of hind foot missteps (footslips) the mouse made was recorded when the mouse crossed the beam a third time. Mice then sat undisturbed for at least 1 h prior to testing. For ethanol-induced ataxia, mice were given an acute injection of 1.5 g/kg ethanol [19] and placed into a holding cage. Ten minutes after the ethanol injection, the mouse was tested on the balance beam and the number of footslips were counted as the mouse crossed the beam by an experimenter blind of the animal’s genotype. If an animal stopped crossing the beam during the training or test session, its tail was gently pinched to encourage movement. If an animal fell from the beam, it was placed back on the beam and allowed to finish crossing [27]. The primary dependent variable analyzed was the number of footslips.

### Dowel

Mice tested on the balance beam were left undisturbed for 1 week prior to undergoing testing for ataxia using the dowel test [28]. The dowel consisted of a 15 mm wooden dowel, elevated 36 cm above the floor. Mice were trained to stay on the dowel for at least 2 min. To meet criteria, this had to be achieved in 5 trials. Following training, mice were left undisturbed to acclimate to the testing room for at least 1 h. Mice then received an injection of 1.5 g/kg ethanol (i.p.) and were immediately placed on the dowel for testing. The latency to fall was recorded with a maximum duration of 300 s. The test was repeated 30 min later. The primary dependent variable was latency to fall (seconds).

### LORR

The loss of righting reflex (LORR) was used to measure the sedative-hypnotic effects of ethanol. Mice were tested for LORR 1 week after the dowel test using previously published methods [19, 29]. Briefly, mice were challenged with an acute 4.1 g/kg ethanol injection (i.p). Upon receiving the injection, the mice were placed into a holding cage until they appeared intoxicated (approximately 1 min). Once intoxicated, the animal was placed on its back in a plexiglass V-shaped trough. Mice were deemed to have lost their righting reflex if they remained on their back for at least 30 s. The experimenter then observed the mice until they turned over onto all four paws—defined as righting themself. Once the animal righted itself it was return to its back. The mouse was deemed to have regained its righting reflex when it was able to right itself 3 times in 1 min.

### Ethanol metabolism

A standard blood ethanol metabolism procedure [17, 24] was done to determine the impact of the *Chrnb4* gene on ethanol metabolism. Mice previously used for ataxia and LORR testing were allowed to rest for 1 week prior to being tested for ethanol metabolism. All mice were challenged with a 3 g/kg ethanol injection (i.p.). Upon receiving the injection, mice were placed into individual holding cages and 10-µl blood samples were taken from the tail vein at 30, 60, 120 and 180 min. BEC were measured using an enzymatic assay as previously described [24]. Briefly, this assay couples the conversion of ethanol to acetaldehyde with the conversion of nicotinamide adenine dinucleotide (NAD) to its reduced form NADH by using alcohol dehydrogenase. The produced NADH is then quantified by a spectrophotometer.

### Statistical analysis

Primary dependent variables examined were ethanol consumption, ethanol preference, total volume consumed, footslips, latency to fall, duration of LORR, and BEC. Independent factors included: genotype, sex, ethanol concentration, and time. A repeated measures analysis of variance (ANOVA) was used to analyze data from the 2-bottle choice ethanol consumption and metabolism studies. Factorial ANOVAs were used to analyze balance beam and LORR data. Due to the maximum duration on the dowel test, these data were not normally distributed, thus they were analyzed with the Kruskal–Wallis test. α < 0.05 was considered significant.

## Results

### Ethanol consumption

**Fig. 1:**
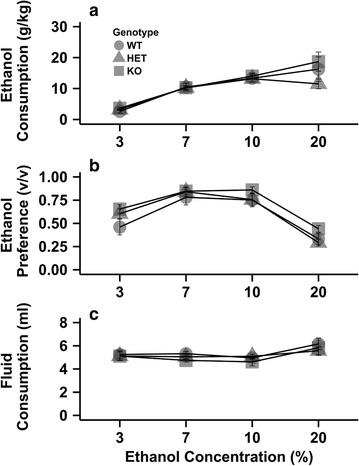
Deletion of the *Chrnb4* gene does not affect ethanol consumption. Data (mean ± SEM) represent average 24 h ethanol consumption (**a**), ethanol preference (**b**), and total fluid consumption (**c**). *Female* WT = 9, HET = 9, KO = 7

The *Chrnb4* gene did not influence choice ethanol consumption (Fig. 1). Ethanol consumption, preference, and total fluid intake varied as a factor of ethanol concentration, but there were no significant main effects of strain or strain × concentration interactions observed. When we analyzed ethanol consumption, there was a significant main effect of concentration (F_3,66_ = 37.1, p < 0.05). Ethanol consumption increased as the concentration rose to 10%, but leveled off after this concentration (all p < 0.05; Fig. 1a). No interaction between concentration and strain for ethanol consumption was detected. A significant main effect of concentration (F_3,66_ = 74.7, p < 0.05) was detected for ethanol preference. There was no interaction between strain and concentration for ethanol preference. Ethanol preference showed an inverted U-shaped pattern (Fig. 1b). Preference increased from 3 to 7%, remaining constant between 7 and 10% and then decreased at 20% (all p < 0.001). When total fluid consumption was examined there was a significant main effect of concentration (F_3,66_ = 8.9, p < 0.05). Mice drank significantly more fluid at 20% compare to all other concentrations (all p < 0.05; Fig. 1c). No other significant effects were observed. All behavioral data split and graphed by sex can be seen in the manuscript’s additional files (see Additional file 2).

### Tastant consumption

**Fig. 2:**
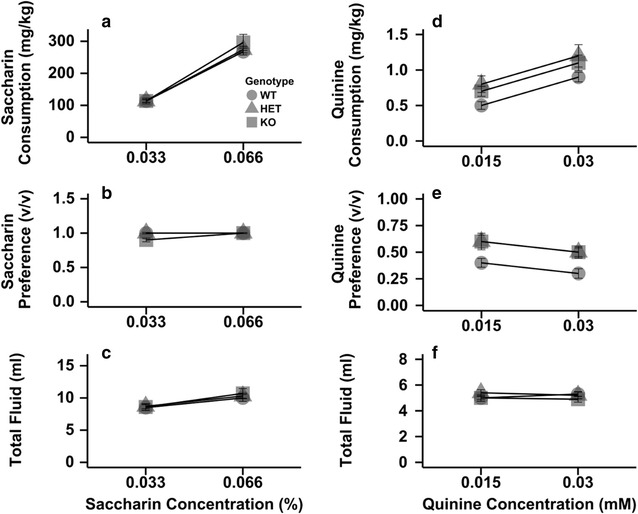
Deletion of the *Chrnb4* gene does not modulate consumption of saccharin or quinine. Data (mean ± SEM) represent average 24 h saccharin consumption (**a**), saccharin preference (**b**), total fluid consumption during saccharin availability (**c**), quinine consumption (**d**), quinine preference (**e**), total fluid consumption during quinine availability (**f**). *Female* WT = 9, HET = 9, KO = 7

To determine if the *Chrnb4* gene influenced taste sensitivity, we examined consumption of a sweet (saccharin) and bitter (quinine) noncaloric tastant (Fig. 2). Consumption, preference, and total fluid intake varied as a factor of saccharin or quinine concentration, but no significant main effects of strain or strain x concentration interactions were observed. A significant main effect of concentration (F_1,22_ = 531.5, p < 0.05) on saccharin consumption was observed. Saccharin consumption increased as the concentration rose to from 0.033 to 0.066% (Fig. 2a; mean ± SEM: 113.5 ± 3.2 vs. 279.3 ± 8.8, respectively). Preference for the saccharin-containing solution increased as the concentration of saccharin increased evidenced by a significant main effect of concentration (F_1,22_ = 5.6, p < 0.05, 0.033%: 0.94 ± 0.01 vs. 0.066%: 0.97 ± 0.01). There was no significant main effect of strain or interaction between strain × concentration for saccharin preference (Fig. 2b). There was a significant main effect of concentration on total fluid consumption (F_1,22_ = 87.0, p < 0.05). Mice drank significantly more fluid when 0.066% saccharin was available (0.033%: 8.5 ± 0.3, 0.066%: 10.3 ± 0.3) (Fig. 2c).

Consumption of quinine also was not influenced by *Chrnb4* genotype (Fig. 2d–f). When quinine consumption was analyzed, there was a significant main effect of concentration (F_1,22_ = 45.2, p < 0.05) for quinine consumption, but no other significant main effects or interactions. Mice consumed significantly less quinine when 0.015 mM quinine was available compared to when 0.03 mM quinine was offered (0.7 ± 0.1 vs. 1.0 ± 0.1, respectively) (Fig. 2d). Concentration of quinine had a significant main effect on preference (F_1,22_ = 13.2, p < 0.05), but neither the main effect of strain nor the strain X concentration interaction was significant (Fig. 2e). Preference decreased as the quinine concentration increased (0.5 ± 0.03 vs. 0.4 ± 0.03, respectively). No significant main effects or interactions were detected when total fluid consumption was examined (Fig. 1f).

### DID

**Fig. 3:**
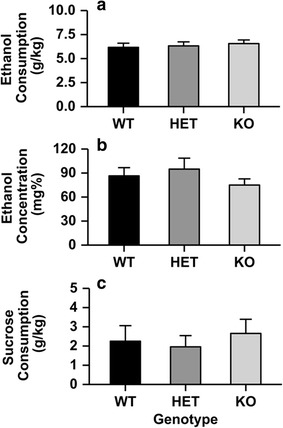
Deletion of the *Chrnb4* gene does not modulate binge-like ethanol consumption. Data (mean ± SEM) represent 4 h ethanol consumption (**a**), BEC after 4 h ethanol intake (**b**), and 4 h sucrose consumption (**c**). *Female* WT = 6, HET = 7, KO = 6; *Male* WT = 5, HET = 6, KO = 4

Deletion of the *Chrnb4* gene did not influence ethanol or sucrose consumption in the DID paradigm (Fig. 3). Data from the DID procedure were analyzed for the final 4 h ethanol or sucrose session [30]. Additionally, the blood ethanol concentration at the end of the 4 h session was analyzed. All 3 dependent variables were analyzed using a 2-way ANOVA with sex and strain included as independent variables. There were no significant main effects or interactions observed for any dependent variable.

### Balance beam

**Fig. 4:**
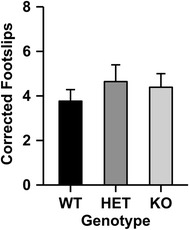
Deletion of the *Chrnb4* gene does not modulate ethanol-induced ataxia measured on the balance beam. Data (mean ± SEM) represent corrected footslips (ethanol slips–baseline slips). *Female* WT = 9, HET = 8, KO = 10; *Male* WT = 8, HET = 7, KO = 8

Deletion of the β4 subunit did not influence baseline or ethanol-induced ataxia measured on the balance beam (Fig. 4). One male HET animal was excluded from the analysis because he dragged his legs on the beam rather than walking on his paws following the ethanol injection. There were no significant effects or interactions on baseline footslips (WT: 1.71 ± 0.37, HET: 1.50 ± 0.33, KO: 1.28 ± 0.25). Therefore, ethanol footslips were corrected by the number of baseline footslips made by each animal [19]. A 2-way ANOVA was used to analyze this corrected score with sex and genotype included as independent factors. No significant main effects or interactions were observed.

### Dowel

**Fig. 5:**
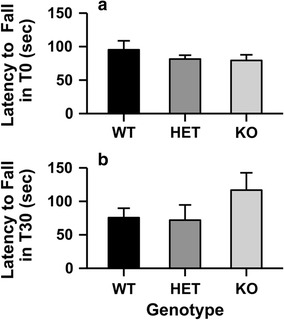
Deletion of the *Chrnb*4 gene did not influence ethanol-induced ataxia measured on the dowel test. Data (mean ± SEM) represent the latency to fall from the dowel (**a**) immediately or (**b**) 30 min after a 1.5 g/kg ethanol injection. *Female* WT = 9, HET = 8, KO = 10; *Male* WT = 8, HET = 7, KO = 8

Similar to results with the balance beam, deletion of the *Chrnb4* gene also did not influence ataxia as measured on the dowel test (Fig. 5). Two mice did not reach training criteria (2 min on the dowel; 1 HET, 1 KO) and were excluded from ethanol testing. There were no statistically significant differences between β4 WT, HET, or KO mice immediately following the ethanol injection or 30 min later on the dowel test when analyzed with a Kruskal–Wallis test.

### LORR

**Fig. 6:**
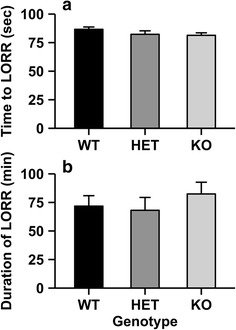
Deletion of the *Chrnb4* gene does not modulate ethanol’s sedative-hypnotic effects as measured by LORR. Data (mean ± SEM) represent time to LORR (**a**) and duration of LORR (**b**). *Female* WT = 9, HET = 8, KO = 10; *Male* WT = 8, HET = 7, KO = 8

Deletion of the *Chrnb4* gene does not influence the sedative-hypnotic effects of ethanol (Fig. 6). Two dependent variables were examined: the time to achieve LORR and duration of LORR. One HET animal did achieve LORR within 3 min of the injection, and was excluded from testing due to a misplaced injection [31]. Two-way ANOVA was used to analyze both dependent variables with sex and genotype included as independent factors. There were no significant main effects or interactions observed for either time to achieve LORR or duration of LORR.

### Metabolism

**Fig. 7:**
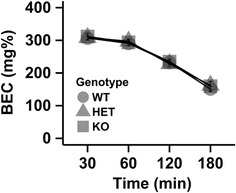
Metabolism of an acute injection of ethanol (3 g/kg) is not influence by the *Chrnb4* gene. Data (mean ± SEM) represent blood ethanol concentrations (BEC). *Female* WT = 9, HET = 8, KO = 10; *Male* WT = 8, HET = 6, KO = 8

Ethanol metabolism was not influenced by the absence of the *Chrnb4* gene (Fig. 7). BEC from 30, 60 120, and 180 min after an acute 3 g/kg ethanol injection was examined with a repeated measures ANOVA. A significant main effect of time (F_3, 129_ = 420.2, p < 0.001) was observed, but no other significant main effects or interactions. As expected BEC levels decreased from the time of injection. Importantly, genotype did not influence ethanol metabolism. Raw data of all experiments are found in supplementary materials (Additional file 3).

## Discussion

Over the last decade, the role of nicotinic acetylcholine receptors in ethanol responses has received substantial attention. Encouraged by results obtained from transgenic mice overexpressing *Chrna5*, *Chrna3*, and *Chrnb4* [13] and pharmacological manipulations of the α3β4 receptors [14], we set out to determine the impact of the β4 nicotinic acetylcholine receptor subunit in ethanol behaviors. Utilizing mice genetically modified to lack the β4 subunit, we chose to examine the role of this subunit in multiple behavioral responses: ethanol consumption, ataxia, and sedation. We found no evidence of the involvement of this subunit in these behaviors. However, it remains possible that other phenotypic responses to ethanol are modulated by this receptor subunit.

The role of the β4 subunit in alcohol-related behaviors has not been well characterized. Two studies have revealed differences in *Chrnb4* mRNA expression in mouse models related to alcohol. The first study was conducted in the FAST and SLOW mouse lines bred for their locomotor response to an acute ethanol injection [32, 33]. SLOW mice that show minimal stimulation when treated with ethanol had greater expression of *Chrnb4* mRNA compared to FAST mice that are sensitive to ethanol-induced locomotor stimulation [34]. Moreover, C57BL/6J mice who consume large amounts of alcohol have reduced *Chrnb4* gene expression compared to DBA/2J animals [35]. Although these genetically defined models likely have many other differences, these data suggested that the *Chrnb4* subunit may influence ethanol behavioral responses. In fact, from these data one might predict that mice with reduced β4 gene expression may consume larger amounts of ethanol compared to those with greater gene expression. Consistent with this hypothesis, transgenic mice that overexpressed the β4, α5, and α3 subunits had reduced ethanol consumption [13]. Unfortunately, we found no evidence for a role of the β4 subunit in ethanol consumption in this study. There are two potential biological mechanisms by which β4-containing acetylcholine receptors could alter ethanol consumption. First, the β4 subunit is expressed in the interpeduncular nucleus and medial habenula [5] and others have suggested that the involvement of these brain structures in ethanol consumption should be investigated [36]. Alternatively, α3β4 nicotinic receptors are also expressed in the chorda tympani taste nerve, and have recently been shown to inhibit ethanol-induced neuronal firing [37]. Given the inconsistent results between our study and others, the involvement of this nicotinic receptor subunit in ethanol consumption warrants further investigation, but the location of β4-containing receptors in the interpeduncular nucleus/medial habenula and chorda tympani taste nerve are two potential areas that could influence this behavior.

It is possible that different results may have been observed using another ethanol-drinking paradigm. For example, the DID procedure results in an intoxicating “binge” of ethanol. Thus, this paradigm may result in maximal ethanol consumption and we would not have the ability to observe increased intake in this model (although other groups have observed increased consumption above that of a C57BL/6J animal in this paradigm [38]). The 2-bottle choice model does not have this same limitation, but unfortunately we were only able to test female mice (due to limited availability of male mice) in this paradigm. Future research incorporating a micro-scale (i.e. lick-o-meter) analysis of drinking behavior may be useful to examine differences in acquisition or drinking-bout intensity/duration that would be obscured in the models we tested.

The gene encoding the β4 nicotinic subunit resides on human chromosome 15 (15q25)/mouse chromosome 9 in a cluster of acetylcholine receptor subunit genes. In addition to *Chrnb4, Chrna5* and *Chrna3* also map to this region. The importance of this gene region in alcohol consumption has been shown through both human and animal studies. Human genetic studies have reported significant associations with variants in this region and initiation of alcohol consumption [8] and use of alcohol use dichotomously defined as those who consume at least one drink per week compared to individuals who drink less or abstain [11]. Similar to the animal model work mentioned above, variants in this region have been implicated in altering gene expression [39, 40]. Pharmacological manipulation of α3β4 nicotinic receptors has also demonstrated the importance of these receptors in ethanol self-administration [14]. Although there is strong support for this gene cluster in ethanol behaviors, findings presented here suggest that these behaviors are not driven by *Chrnb4*. Further, these lead to the hypothesis that the *Chrna3* subunit may mediate ethanol consumption. Although this subunit has been shown to influence in ethanol-induced locomotor stimulation [41], we know of no data on ethanol consumption in mice lacking this subunit (homozygote *Chrna3* knock-outs do not survive).

Nicotinic receptors have been implicated in a number of ethanol behaviors. Pharmacologic manipulation of nicotinic receptors have demonstrated their involvement in: ethanol consumption [42], ataxia [43], sedation [26], locomotion [34, 44], reward [45], and withdrawal [46, 47]. Moreover, the use of genetically modified animals has yielded additional information on which receptor subunits are important in these behaviors. For example, *Chrna5* [48], *Chrna6* [19], and *Chrna7* [49] and have all been implicated in the sedative properties of ethanol. Interestingly, *Chrna5* [48] and *Chrna6* [19] do not have accompanying changes in ethanol consumption, but *Chrna7* does [17]. These data highlight the importance of examining the role of different nicotinic acetylcholine receptors for a range of behaviors.

One behavior not examined in this paper is ethanol withdrawal. Both pharmacological and genetic manipulations have shown that nicotinic receptors are important for this behavior [46, 47, 50]. Recently, Perez and colleagues have shown that nicotinic acetylcholine receptors in the medial habenula are important in ethanol withdrawal [47]. Although this study did not examine the specific nicotinic receptors that mediate this response, α3β4 receptors are known to be abundant in this region [5]. Moreover the β4 subunit has been implicated in nicotine withdrawal [51], thus future studies should examine the role of this subunit in alcohol withdrawal.

This study is not without important limitations. For example, it is important to note the limitations of the knockout model. The mice we tested have passed through development in the absence of the β4 subunit, so it is possible another subunit (e.g. β2) may compensate for its loss. Additionally, we report no significant differences with our behaviors of interest and the usefulness of this deletion could be in question. However, studies with nicotine behaviors using knockout animals generated from the same mouse colony and tested contemporaneously to this study have reported differences in nicotine tolerance [52].

## Conclusions

Although these results do not support a role of *Chrnb4* in alcohol-related behaviors they provide critical information to the field. There are important known genetic associations between variants in the *CRHNA5*/*CHRNA3*/*CHRNB4* gene cluster and alcohol-related behaviors. Yet because these genes are found in a cluster it makes it difficult to determine which subunit drives these effects. Animal models allow us to separate the influence of each gene. Thus, these results inform us of the role of *Chrnb4* in alcohol-related behaviors. These data provide evidence that this receptor subunit may not influence alcohol consumption, ataxia or sedation, but further work examining the involvement of this gene in ethanol withdrawal and locomotor stimulation may be warranted.

## Additional files

**Additional file 1.** Supplementary data.

**Additional file 2.** Graphs by sex.

**Additional file 3.** Raw experimental data.

## Abbreviations

WT: wildtype
HET: heterozygous
KO: knockout
DID: drinking-in-the-dark
LORR: loss of righting reflex
BEC: blood ethanol concentration
ANOVA: analysis of variance

## References

[1] Grant BF, Goldstein RB, Saha TD (2015). Epidemiology of dsm-5 alcohol use disorder: results from the national epidemiologic survey on alcohol and related conditions III. JAMA Psychiatry.

[2] Hingson R, Rehm J (2014). Measuring the burden: alcohol’s evolving impact. Alcohol Res Curr Rev.

[3] Rahman S (2013). Nicotinic receptors as therapeutic targets for drug addictive disorders. CNS Neurol Disord Drug Targets.

[4] Wu J, Gao M, Taylor DH (2014). Neuronal nicotinic acetylcholine receptors are important targets for alcohol reward and dependence. Acta Pharmacol Sin.

[5] Zoli M, Pistillo F, Gotti C (2015). Diversity of native nicotinic receptor subtypes in mammalian brain. Neuropharmacology.

[6] Gotti C, Zoli M, Clementi F (2006). Brain nicotinic acetylcholine receptors: native subtypes and their relevance. Trends Pharmacol Sci.

[7] Millar NS, Gotti C (2009). Diversity of vertebrate nicotinic acetylcholine receptors. Neuropharmacology.

[8] Schlaepfer IR, Hoft NR, Collins AC, Corley RP, Hewitt JK, Hopfer CJ (2008). The CHRNA5/A3/B4 gene cluster variability as an important determinant of early alcohol and tobacco initiation in young adults. Biol Psychiatry.

[9] Joslyn G, Brush G, Robertson M, Smith TL, Kalmijn J, Schuckit M (2008). Chromosome 15q25.1 genetic markers associated with level of response to alcohol in humans. Proc Natl Acad Sci USA.

[10] Choquet H, Joslyn G, Lee A, Kasberger J, Robertson M, Brush G (2013). Examination of rare missense variants in the CHRNA5-A3-B4 gene cluster to level of response to alcohol in the San Diego Sibling Pair study. Alcohol Clin Exp Res.

[11] Hällfors J, Loukola A, Pitkäniemi J, Broms U, Männistö S, Salomaa V (2013). Scrutiny of the CHRNA5-CHRNA3-CHRNB4 smoking behavior locus reveals a novel association with alcohol use in a Finnish population based study. Int J Mol Epidemiol Genet.

[12] Wang J-C, Spiegel N, Bertelsen S, Le N, McKenna N, Budde JP (2013). Cis-regulatory variants affect CHRNA5 mRNA expression in populations of African and European ancestry. PLoS ONE.

[13] Gallego X, Ruiz-Medina J, Valverde O, Molas S, Robles N, Sabrià J (2012). Transgenic over expression of nicotinic receptor alpha 5, alpha 3, and beta 4 subunit genes reduces ethanol intake in mice. Alcohol.

[14] Chatterjee S, Steensland P, Simms JA, Holgate J, Coe JW, Hurst RS (2011). Partial agonists of the α3β4* neuronal nicotinic acetylcholine receptor reduce ethanol consumption and seeking in rats. Neuropsychopharmacology.

[15] Xu W, Orr-Urtreger A, Nigro F, Gelber S, Sutcliffe CB, Armstrong D (1999). Multiorgan autonomic dysfunction in mice lacking the β2 and the β4 subunits of neuronal nicotinic acetylcholine receptors. J Neurosci.

[16] Salminen O, Murphy KL, McIntosh JM, Drago J, Marks MJ, Collins AC (2004). Subunit composition and pharmacology of two classes of striatal presynaptic nicotinic acetylcholine receptors mediating dopamine release in mice. Mol Pharmacol.

[17] Kamens HM, Andersen J, Picciotto MR (2010). Modulation of ethanol consumption by genetic and pharmacological manipulation of nicotinic acetylcholine receptors in mice. Psychopharmacology.

[18] Kamens HM, Burkhart-Kasch S, McKinnon CS, Li N, Reed C, Phillips TJ (2006). Ethanol-related traits in mice selectively bred for differential sensitivity to methamphetamine-induced activation. Behav Neurosci.

[19] Kamens HM, Hoft NR, Cox RJ, Miyamoto JH, Ehringer MA (2012). The α6 nicotinic acetylcholine receptor subunit influences ethanol-induced sedation. Alcohol.

[20] Phillips TJ, Crabbe JC, Metten P, Belknap JK (1994). Localization of genes affecting alcohol drinking in mice. Alcohol Clin Exp Res.

[21] Kamens HM, Miyamoto J, Powers MS, Ro K, Soto M, Cox R (2015). The β3 subunit of the nicotinic acetylcholine receptor: modulation of gene expression and nicotine consumption. Neuropharmacology.

[22] Rhodes JS, Best K, Belknap JK, Finn DA, Crabbe JC (2005). Evaluation of a simple model of ethanol drinking to intoxication in C57BL/6J mice. Physiol Behav.

[23] Rhodes JS, Ford MM, Yu C-H, Brown LL, Finn DA, Garland T (2007). Mouse inbred strain differences in ethanol drinking to intoxication. Genes Brain Behav..

[24] Ehringer MA, Hoft NR, Zunhammer M (2009). Reduced alcohol consumption in mice with access to a running wheel. Alcohol.

[25] Kamdar NK, Miller SA, Syed YM, Bhayana R, Gupta T, Rhodes JS (2007). Acute effects of naltrexone and GBR 12909 on ethanol drinking-in-the-dark in C57BL/6J mice. Psychopharmacology.

[26] Kamens HM, Andersen J, Picciotto MR (2010). The nicotinic acetylcholine receptor partial agonist varenicline increases the ataxic and sedative-hypnotic effects of acute ethanol administration in C57BL/6J mice. Alcohol Clin Exp Res.

[27] Crabbe JC, Metten P, Yu C-H, Schlumbohm JP, Cameron AJ, Wahlsten D (2003). Genotypic differences in ethanol sensitivity in two tests of motor incoordination. J Appl Physiol.

[28] Crabbe JC, Cotnam CJ, Cameron AJ, Schlumbohm JP, Rhodes JS, Metten P (2003). Strain differences in three measures of ethanol intoxication in mice: the screen, dowel and grip strength tests. Genes Brain Behav.

[29] Crabbe JC, Metten P, Ponomarev I, Prescott CA, Wahlsten D (2006). Effects of genetic and procedural variation on measurement of alcohol sensitivity in mouse inbred strains. Behav Genet.

[30] Marshall SA, Casachahua JD, Rinker JA, Blose AK, Lysle DT, Thiele TE (2016). IL-1 receptor signaling in the basolateral amygdala modulates binge-like ethanol consumption in male C57BL/6 J mice. Brain Behav Immun.

[31] Ponomarev I, Crabbe JC (2002). A novel method to assess initial sensitivity and acute functional tolerance to hypnotic effects of ethanol. J Pharmacol Exp Ther.

[32] Phillips TJ, Burkhart-Kasch S, Terdal ES, Crabbe JC (1991). Response to selection for ethanol-induced locomotor activation: genetic analyses and selection response characterization. Psychopharmacology.

[33] Shen EH, Harland RD, Crabbe JC, Phillips TJ (1995). Bidirectional selective breeding for ethanol effects on locomotor activity: characterization of FAST and SLOW mice through selection generation 35. Alcohol Clin Exp Res.

[34] Kamens HM, Phillips TJ (2008). A role for neuronal nicotinic acetylcholine receptors in ethanol-induced stimulation, but not cocaine- or methamphetamine-induced stimulation. Psychopharmacology.

[35] Symons MN, Weng J, Diehl E, Heo E, Kleiber ML, Singh SM (2010). Delineation of the role of nicotinic acetylcholine receptor genes in alcohol preference in mice. Behav Genet.

[36] Hendrickson LM, Guildford MJ, Tapper AR (2013). Neuronal nicotinic acetylcholine receptors: common molecular substrates of nicotine and alcohol dependence. Front Psychiatry.

[37] Ren ZJ, Mummalaneni S, Qian J, Baumgarten CM, DeSimone JA, Lyall V (2015). Nicotinic acetylcholine receptor (nAChR) dependent chorda tympani taste nerve responses to nicotine, ethanol and acetylcholine. PLoS ONE.

[38] Phillips TJ, Reed C, Burkhart-Kasch S, Li N, Hitzemann R, Yu C-H (2010). A method for mapping intralocus interactions influencing excessive alcohol drinking. Mamm Genome Off J Int Mamm Genome Soc..

[39] Flora AV, Zambrano CA, Gallego X, Miyamoto JH, Johnson KA, Cowan KA (2013). Functional characterization of SNPs in CHRNA3/B4 intergenic region associated with drug behaviors. Brain Res.

[40] Gallego X, Cox RJ, Laughlin JR, Stitzel JA, Ehringer MA (2013). Alternative CHRNB4 3′-UTRs mediate the allelic effects of SNP rs1948 on gene expression. PLoS ONE.

[41] Kamens HM, McKinnon CS, Li N, Helms ML, Belknap JK, Phillips TJ (2009). The alpha 3 subunit gene of the nicotinic acetylcholine receptor is a candidate gene for ethanol stimulation. Genes Brain Behav.

[42] Hendrickson LM, Zhao-Shea R, Tapper AR (2009). Modulation of ethanol drinking-in-the-dark by mecamylamine and nicotinic acetylcholine receptor agonists in C57BL/6J mice. Psychopharmacology.

[43] Taslim N, Al-Rejaie S (2008). Saeed Dar M. Attenuation of ethanol-induced ataxia by alpha(4)beta(2) nicotinic acetylcholine receptor subtype in mouse cerebellum: a functional interaction. Neuroscience.

[44] Larsson A, Svensson L, Söderpalm B, Engel JA (2002). Role of different nicotinic acetylcholine receptors in mediating behavioral and neurochemical effects of ethanol in mice. Alcohol.

[45] Bhutada P, Mundhada Y, Ghodki Y, Dixit P, Umathe S, Jain K (2012). Acquisition, expression, and reinstatement of ethanol-induced conditioned place preference in mice: effects of exposure to stress and modulation by mecamylamine. J Psychopharmacol Oxf Engl.

[46] Bhutada PS, Mundhada YR, Bansod KU, Umathe SN, Kahale VP, Dixit PV (2010). Inhibitory influence of mecamylamine on ethanol withdrawal-induced symptoms in C57BL/6J mice. Behav Pharmacol.

[47] Perez E, Quijano-Cardé N, De Biasi M (2015). Nicotinic mechanisms modulate ethanol withdrawal and modify time course and symptoms severity of simultaneous withdrawal from alcohol and nicotine. Neuropsychopharmacology.

[48] Santos N, Chatterjee S, Henry A, Holgate J, Bartlett SE (2013). The α5 neuronal nicotinic acetylcholine receptor subunit plays an important role in the sedative effects of ethanol but does not modulate consumption in mice. Alcohol Clin Exp Res.

[49] Bowers BJ, McClure-Begley TD, Keller JJ, Paylor R, Collins AC, Wehner JM (2005). Deletion of the alpha7 nicotinic receptor subunit gene results in increased sensitivity to several behavioral effects produced by alcohol. Alcohol Clin Exp Res.

[50] Butt CM, King NM, Stitzel JA, Collins AC (2004). Interaction of the nicotinic cholinergic system with ethanol withdrawal. J Pharmacol Exp Ther.

[51] Salas R, Pieri F, De Biasi M (2004). Decreased signs of nicotine withdrawal in mice null for the beta4 nicotinic acetylcholine receptor subunit. J Neurosci.

[52] Meyers EE, Loetz EC, Marks MJ (2015). Differential expression of the beta4 neuronal nicotinic receptor subunit affects tolerance development and nicotinic binding sites following chronic nicotine treatment. Pharmacol Biochem Behav.

