# Supplementary material for: No evidence of a role of the β4 subunit of the nicotinic acetylcholine receptor in alcohol-related behaviors
Source: BMC Res Notes. 2017 Apr 5;10:151. doi: 10.1186/s13104-017-2470-7 (PMC5382442; doi:10.1186/s13104-017-2470-7)
Supplement: Supplementary file 1 — Additional file 1. Supplementary data. [file 13104_2017_2470_MOESM1_ESM.pdf]

Developmental stage and weight of the animals tested at the beginning of each experiment.

2-Bottle Choice Ethanol Consumption & 2-Bottle Choice Tastant Consumption:

Genotype	Mean Age	Age Range	Mean Weight	Weight Range
**WT**	84.9	65 – 102	21.7	17.3 – 25.2
**HET**	85.0	65 – 102	22.2	20.4 – 26.2
**KO**	89.7	65 – 105	21.3	19.8 – 22.9

Drinking in the Dark (DID):

Genotype	Mean Age	Age Range	Mean Weight	Weight Range
**WT**	107.5	86 – 144	25.9	22.3 – 31.0
**HET**	99.3	78 – 144	25.8	22.2 – 29.7
**KO**	93.7	80 – 102	25.5	21.6 – 32.6

Balance Beam, Dowel, LORR, and Metabolism:

Genotype	Mean Age	Age Range	Mean Weight	Weight Range
**WT**	102.6	70 – 127	25.9	21.7 – 35.0
**HET**	96.3	72 – 127	25.9	21.1 – 32.8
**KO**	98. 6	72 – 127	25.6	18.8 – 34.0
