# Supplementary material for: No evidence of a role of the β4 subunit of the nicotinic acetylcholine receptor in alcohol-related behaviors
Source: BMC Res Notes. 2017 Apr 5;10:151. doi: 10.1186/s13104-017-2470-7 (PMC5382442; doi:10.1186/s13104-017-2470-7)
Supplement: Supplementary file 2 — Additional file 2. Graphs by sex. [file 13104_2017_2470_MOESM2_ESM.pdf]

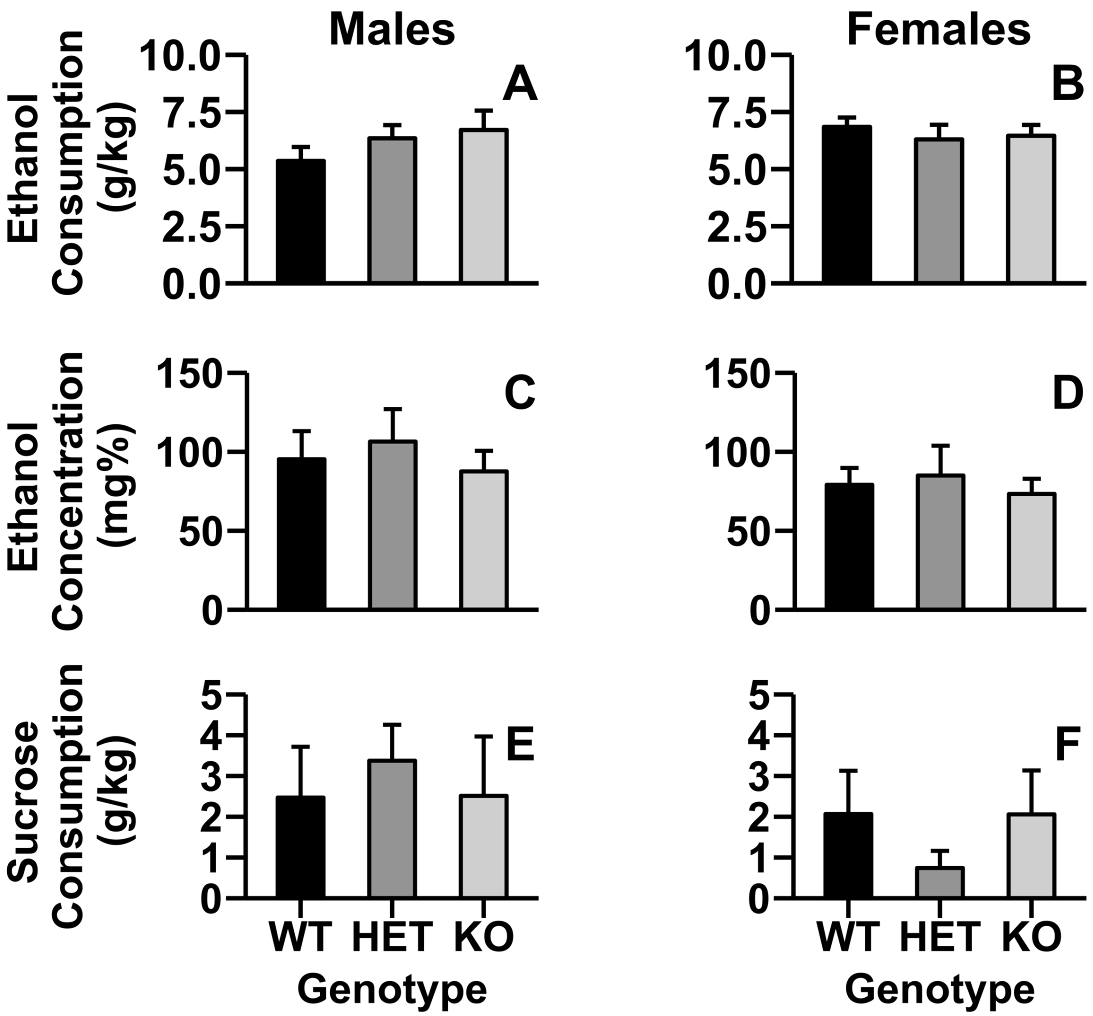

Binge-like ethanol consumption in male and female *Chrnb4* mice. Data (mean ± SEM) represent 4 h ethanol consumption in male (A) and female (B) animals, blood ethanol concentration (BEC) after 4 h ethanol intake in male (C) and female (D) animals, and 4 h sucrose consumption in male (E) and female (F) animals.

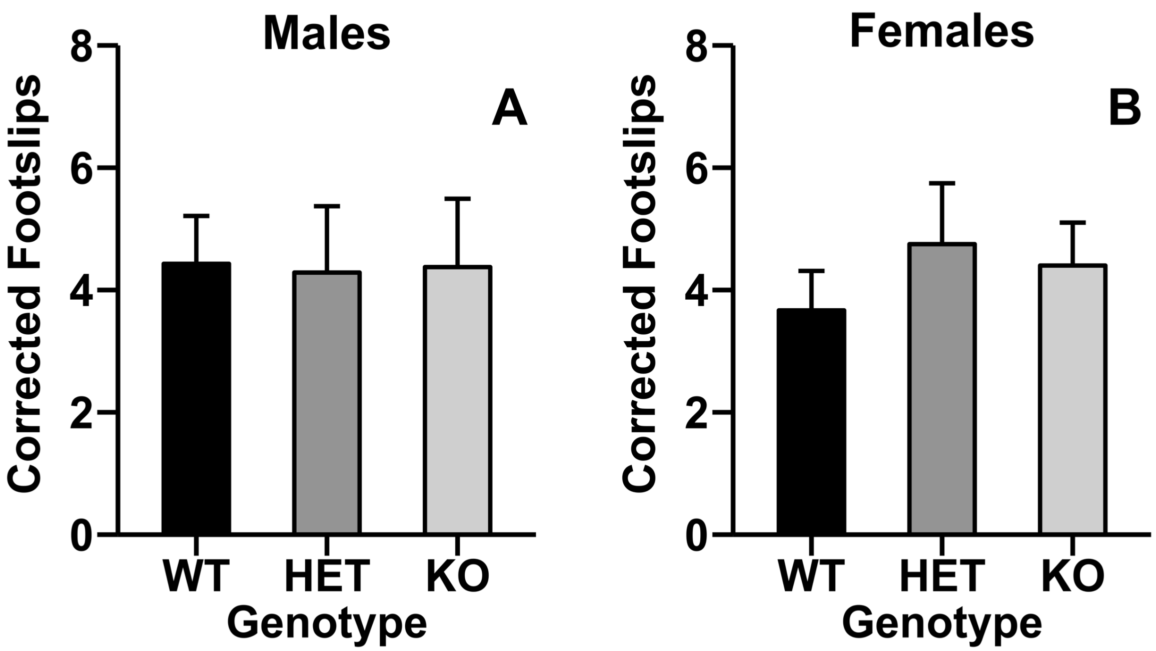

Ethanol-induced ataxia measured on the balance beam in male and female mice.

Data (mean ± SEM) represent corrected footslips (ethanol slips – baseline slips) in male (A) and female (B) *Chrnb4* mice.

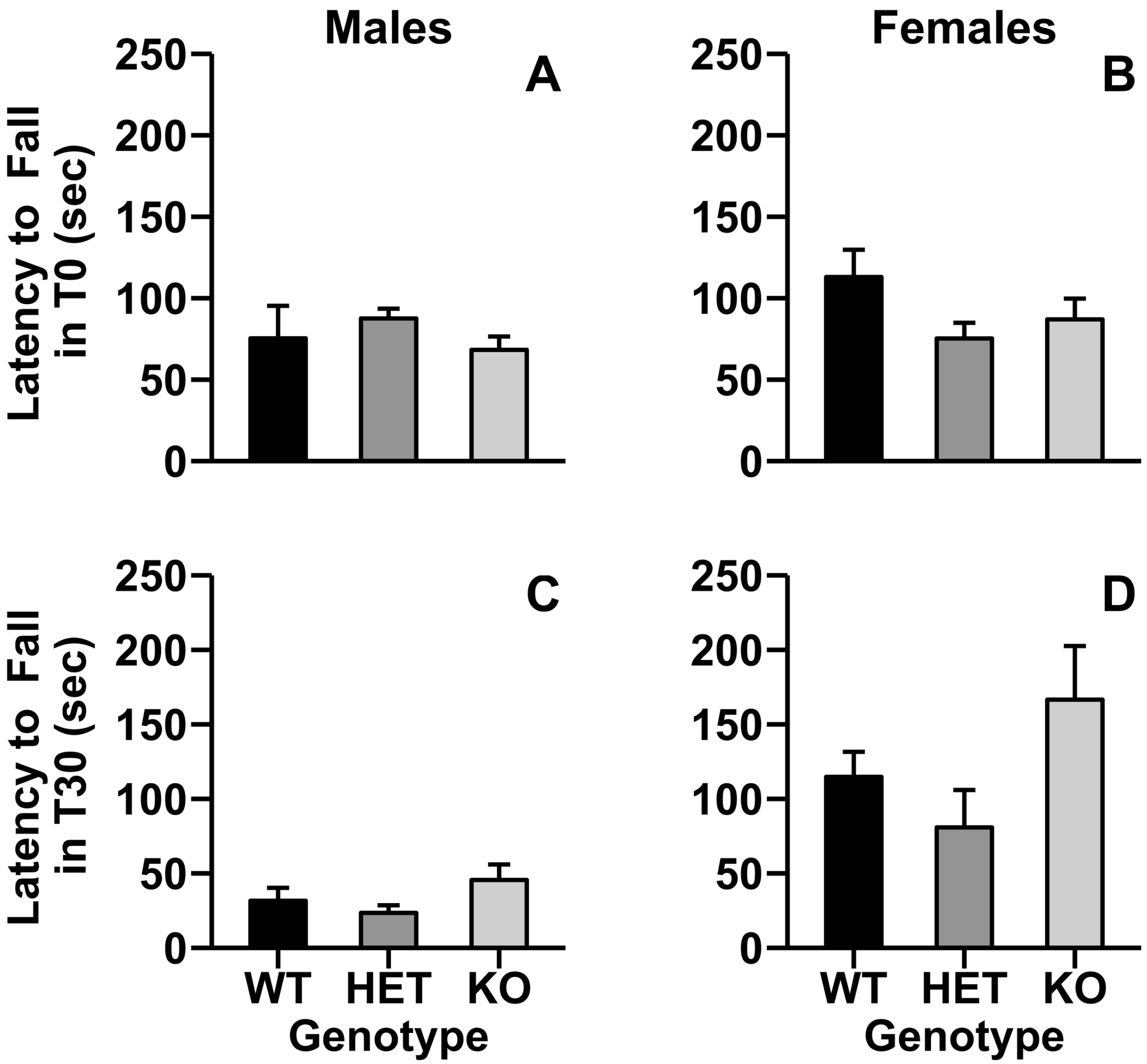

Ethanol-induced ataxia measured on the dowel test in male and female *Chrnb4* mice. Data (mean ± SEM) represent the latency to fall from the dowel in male (A) and female (B) mice immediately or (male = C, female = D) 30 minutes after a 1.5 g/kg ethanol injection.

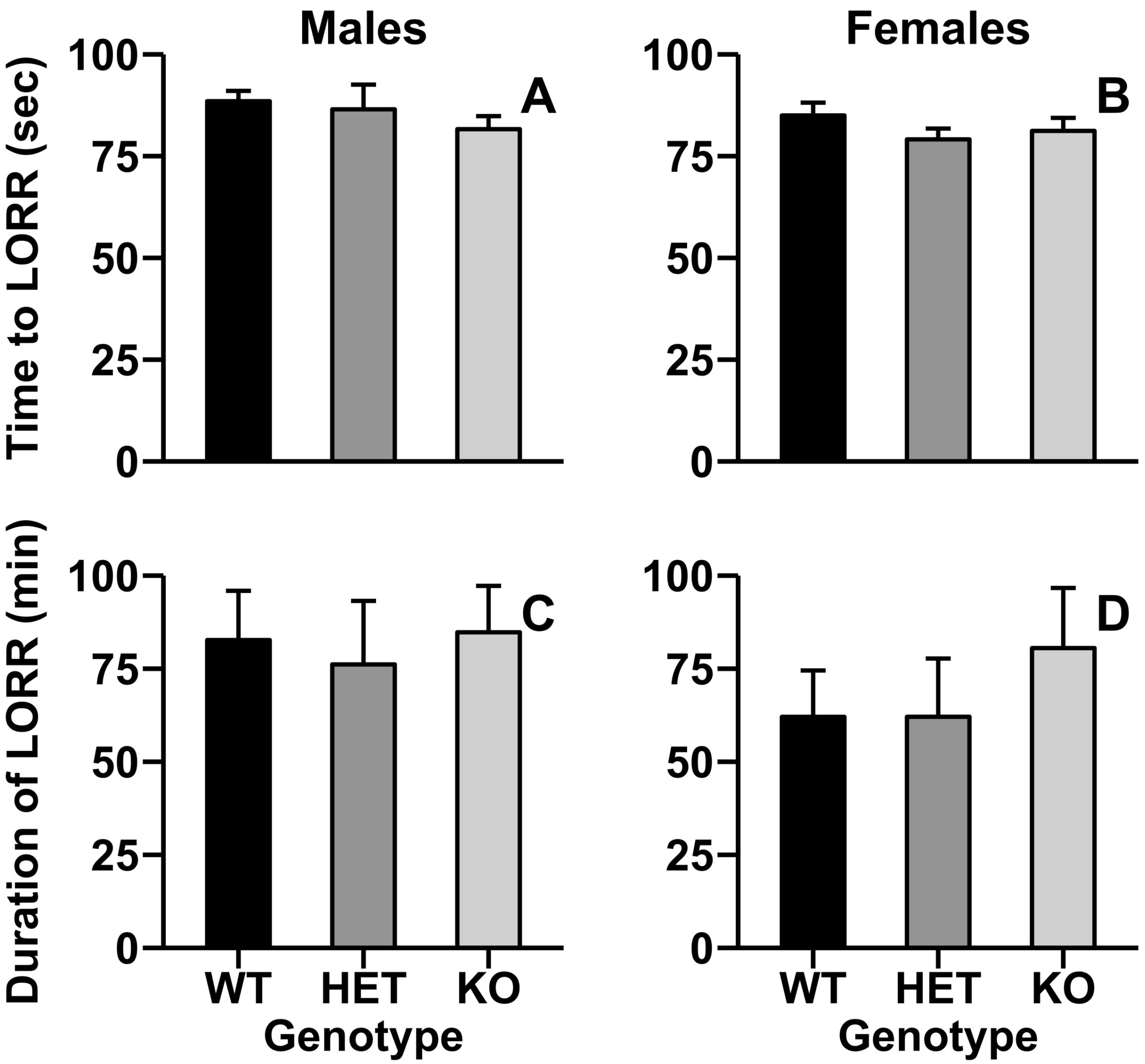

Ethanol-induced sedative-hypnotic effects as measured by LORR in male and female *Chrnb4* mice. Data (mean ± SEM) represent time to LORR in male (A) and female (B) mice and duration of LORR in male (C) and female (D) animals.

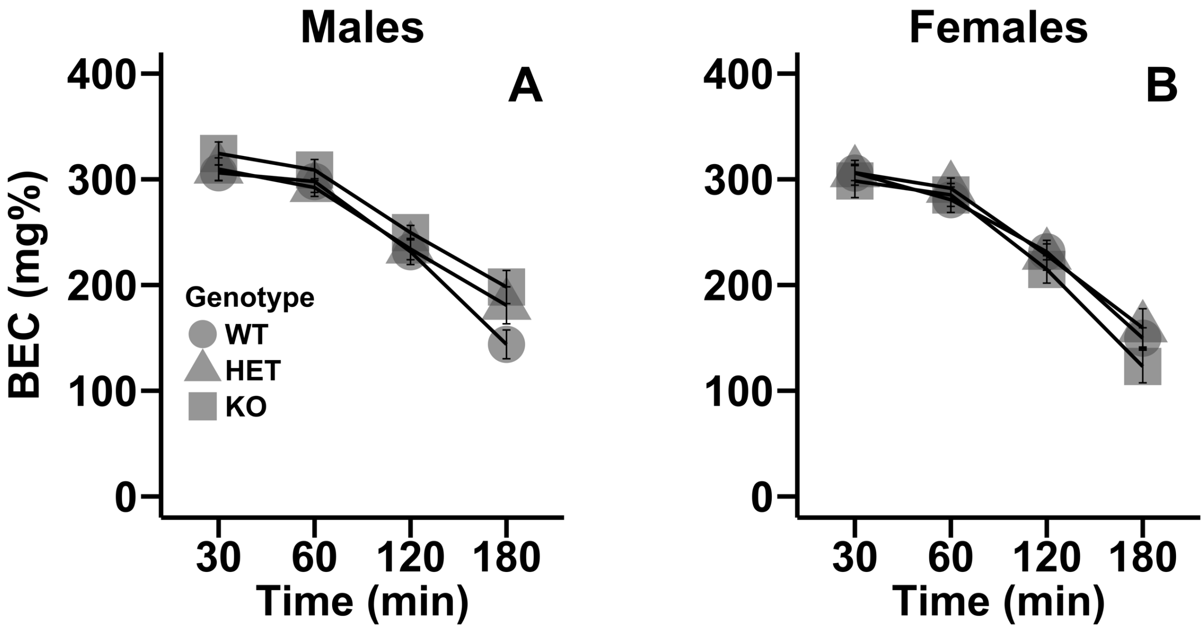

Metabolism of an acute injection of ethanol (3 g/kg) in male and female *Chrnb4* mice. Data (mean ± SEM) represent blood ethanol concentrations (BEC) in male (A) and female (B) mice.
